# Antimalarial compounds exhibit variant- and cell-type-specific activity against SARS-CoV-2 isolated in Panama

**DOI:** 10.3389/fphar.2025.1537053

**Published:** 2025-06-04

**Authors:** Mario Quijada, Marlene Castillo-Bultron, Yamilka Díaz, Yaneth Pitti, Danilo Franco, Carolina De La Guardia, Dalkiria Campos, Eduardo Cornejo, Marlon Núñez, Lariza Mendoza, Sandra López-Vergès, Ariel H. Magallon-Tejada, Nicanor Obaldia

**Affiliations:** ^1^ Departamento de Investigaciones en Parasitología, Centro para la Evaluación de Drogas y Vacunas Antimaláricas, Instituto Conmemorativo Gorgas de Estudios de la Salud, Panamá City, Panama; ^2^ Departamento de Investigación en Virología y Biotecnología, Instituto Conmemorativo Gorgas de Estudios de la Salud, Panamá City, Panama; ^3^ Centro de Biologia Celular y Molecular de Enfermedades, Indicasat-AIP, Panamá, Panamá City, Panama; ^4^ Departamento de Genómica y Proteómica, Instituto Conmemorativo Gorgas de Estudios de la Salud, Panamá City, Panama

**Keywords:** COVID-19, SARS-CoV-2 variants, *in vitro*, antimalarials, antivirals, Vero-E6, Calu-3, Panama

## Abstract

**Background:**

This study evaluates the antiviral activity of antimalarial compounds against SARS-CoV-2 variants isolated in Panama (2020–2022).

**Methods:**

For this purpose, we conducted a series of *in vitro* assays in two host mammalian cell systems, Vero-E6 and Calu-3 cells, to assess the antiviral activity of twenty-six antimalarials and antiviral compounds against the Delta and A2.5 variants.

**Results:**

In the initial screening using Vero-E6 cells, with an antiviral inhibition threshold of ≥20% and cell viability of ≥80%, chloroquine (CQ) significantly inhibited the Delta variant. Meanwhile, amodiaquine (AQ), artemisone (ASO), and ivermectin (IVM) showed activity against the A2.5 variant. In Calu-3 cells, a wider variety of compounds, including chloroquine (CQ), amodiaquine (AQ), artesunate (AS), lumefantrine (LUM), and hydroxychloroquine (HCQ), were found to be effective against the Delta variant. However, only amodiaquine (AQ) and arteether (AE) showed activity against the A2.5 variant, indicating that the response varies depending on the variant and the type of cells involved. Secondary screenings further demonstrated CQ’s high inhibitory activity, with an IC50 of 6.3 μM and a selectivity index of 8, followed by HCQ, which was 1.8 times more potent against A2.5 than Delta. Time-of-addition experiments suggested that CQ and primaquine (PQ) were ineffective during the viral adsorption phase but showed a dose-dependent antiviral effect against the A2.5 variant in the early replication phase, whereas the Delta variant showed resistance.

**Conclusion:**

This study underscores the critical role of selecting appropriate cell models for SARS-CoV-2 research, as drug efficacy varies between viral variants and host cell types.

## 1 Introduction

Since its emergence in December 2019, the severe acute respiratory syndrome virus (SARS-CoV-2), which causes COVID-19, has undergone multiple mutations, resulting in new variants of concern. These variants exhibit increased transmissibility and spread rapidly across different regions of the planet (PAHO, 2023; Ma et al., 2024; Carabelli et al., 2023; Zhou et al., 2023; Yan and Muller, 2021).

Despite the World Health Organization (WHO) declaring in May 2023 that COVID-19 no longer constitutes a public health emergency (PAHO, 2023), the emergence of new variants, some containing escape mutations to antibody neutralization induced by vaccination or previous infections (Ma et al., 2024; Carabelli et al., 2023), has reignited the search for new or repurposed drugs against the virus (Zhou et al., 2023), with particular interest in understanding the antiviral activity or lack thereof of antimalarials and other antiparasitic compounds against SARS-CoV-2 variants. It is noteworthy that this occurs against a backdrop of over 769 million confirmed cases and more than 6.9 million deaths reported worldwide as of 6 August 2023 (WHO, 2023).

Only a few antiviral drugs, such as remdesivir (Yan and Muller, 2021), nirmatrelvir/ritonavir (Paxlovid) (Najjar-Debbiny et al., 2022) and molnupiravir (Khoo et al., 2022), are currently approved by the Food and Drug Administration (FDA) and the European Medicines Agency (EMA) to treat COVID-19. However, researchers are continuing to search for more effective alternatives.

Repurposing drugs is a cost-effective strategy as developing new antiviral agents is a long and expensive process, especially in the rapidly evolving viral variants scenario. Among the first drugs to be repurposed for the treatment of COVID-19 were the antimalarials 4-aminoquinolines, chloroquine (CQ) (Keyaerts et al., 2009; Keyaerts et al., 2004; Wang et al., 2020; Persoons et al., 2021), and its metabolite hydroxychloroquine (HCQ) (Persoons et al., 2021; Yao et al., 2020).

Repurposed drugs exert antiviral effects primarily by inhibiting enzymes critical for viral replication (Mohanty et. al., 2021). For example, camostat mesylate targets the host serine protease TMPRSS2, whereas umifenovir interferes with the viral spike protein. Imatinib mesylate inhibits Abl kinase, and mefloquine hydrochloride acts on angiotensin-converting enzyme 2 (ACE2). The endosomal pH regulators chloroquine and hydroxychloroquine also contribute to viral inhibition. Telaprevir is active against viral proteases, while nelfinavir mesylate inhibits the main protease (Mpro). Itraconazole disrupts oxysterol-binding protein function, and remdesivir inhibits viral RNA-dependent RNA polymerase. Auranofin targets intracellular redox enzymes, and thalidomide functions as an immunomodulatory agent.

CQ, a 4-aminoquinoline, has been in use for more than 70 years as an antimalarial (Obaldia et al., 2018; Obaldia et al., 1997) and, more recently, for its immunomodulatory activity in the treatment of autoimmune diseases such as rheumatoid arthritis and systemic lupus erythematosus (Keyaerts et al., 2004), reducing the inflammatory response –cytokine storm–, one of the hallmark outcomes of COVID-19 (Tang et al., 2020). CQ and HCQ have shown *in vitro* and *in vivo* activity against SARS-CoV-2 (Keyaerts et al., 2009; Keyaerts et al., 2004), although their clinical efficacy remains controversial (Yao et al., 2020; Ho et al., 2021; Zhong et al., 2022; Bauman and Tisdale, 2020; Pillat et al., 2020). Unfortunately, CQ could cause adverse events such as gastrointestinal problems, retinopathy, QT prolongation (Satarker et al., 2020), cardiac toxicity, and even death (Bauman and Tisdale, 2020; Banerjee et al., 2023).

Its mechanism of action is poorly understood, but recent studies have shown that CQ exerts its antiviral effect through different mechanisms, for example, by increasing the endosomal pH required for virus/cell fusion and by interfering with and changing the pattern of the glycosylation of cellular receptors such as the HIV-1 gp120 protein. CQ also acts by inhibiting and interfering with virus infection and replication by affecting autophagy, a mechanism that has an inhibitory effect on viral infections that invade cells through the endosomal route, such as Borna disease virus, avian leukemia virus, Zika, and SARS-CoV (Gao et al., 2020) (Huang and Tang, 2020).

Another 4-aminoquinoline, amodiaquine (AQ) (Si et al., 2021), and the 8-aminoquinolines, primaquine (PQ) (Persoons et al., 2021) and its analog tafenoquine (TQ), approved for radical cure of *Plasmodium vivax* and malaria prophylaxis (Persoons et al., 2021; Chen et al., 2022), have also shown *in vitro* activity, though to a lesser extent, against the SARS-CoV-2 virus.

Similarly, artemisinin (Qinghaosu) (QHS) –a first-line sesquiterpene lactone antimalarial and the active molecule of the *Artemisia annua* plant (*qīnghāo*), used for centuries in traditional Chinese medicine for the treatment of intermittent fevers, malaria, and respiratory infections– (Kapepula et al., 2020) and its semisynthetic derivatives artesunate (AS), arteether (AE), artemether (AM) and artelinic acid (AA), apart from its rapid antimalarial action (Obaldia et al., 2009), have demonstrated *in vitro* activity against SARS-CoV-2 virus (Gendrot et al., 2020a; Zhou et al., 2021), and efficacy in clinical trial against COVID-19 (Shi et al., 2022).

The combination of AS and AQ, apart from its activity against SARS-Cov-2, has been shown to have potent antiviral activity against dengue virus (DENV2) (Boonyasuppayakorn et al., 2014) and West Nile Virus (WNV), as well as in patients infected with the Ebola virus, reducing the risk of mortality (Gignoux et al., 2016). In addition, semisynthetic derivatives of artemisinin have demonstrated activity against parasites, cancer cells, and viruses, such as human cytomegalovirus (HCMV), hepatitis B virus (HBV), and human papillomavirus (HPV), among others, with effective concentrations (EC_50_) that are in the micromolar range, compared to the nanomolar range of other antimalarials (Gignoux et al., 2016). Other antimalarials, such as the quinolinometanol mefloquine (MQ), have demonstrated *in vitro* antiviral activity alone (Fan et al., 2020) or in combination with artemisinin derivatives against SARS-CoV-2 (Gendrot et al., 2020a).

Given the ongoing evolution of SARS-CoV-2 and the limitations of existing treatments, repurposing drugs remains a cost-effective strategy for identifying new antiviral options. Antimalarial and antiparasitic compounds have shown *in vitro* activity against SARS-CoV-2, but their effectiveness across different variants and host cells is poorly understood.

This study compares the *in vitro* antiviral activity of selected antimalarials and other antiparasitic compounds against the Delta and A2.5 variants of SARS-CoV-2 in 2 cell cultures: Vero-E6 and Calu-3. We hypothesize that the *in vitro* activity of antimalarial compounds against the SARS-CoV-2 virus depends on the variant and the host cell type.

## 2 Materials and methods

### 2.1 Virus and cells

To conduct the *in vitro* experiments on the antiviral activity of various compounds, we utilized the SARS-CoV-2 variants A2.5 (M2169), Mu (SEQ83), and Delta (SEQ203), which were isolated at the Instituto Conmemorativo Gorgas de Estudios de la Salud (ICGES) in Panama. All *in vitro* cell infection procedures were performed in a level II security cabinet within a BSL3 laboratory. Virus isolates were replicated, and aliquots were prepared and stored at −80°C until use. African green monkey kidney cells (Vero-E6) (ATCC CRL-1587) and human lung adenocarcinoma epithelial cells (Calu-3) (ATCC HTB-55) were grown and maintained in Minimal Essential Medium (MEM) (Gibco, Life Technologies) supplemented with either 10 or 20% fetal bovine serum (FBS) respectively (Gibco, Life Technologies), 1% penicillin/streptomycin and 50 μg/mL gentamicin (Gibco, Life Technologies). Calu-3 cells were supplemented further with sodium pyruvate solution (Lonza, BioWhittaker, Walkersville, MD, USA). Cell cultures were maintained at 37°C in a humidified 5% CO_2_ atmosphere in a Thermo Scientific Steri-Cycle i160 incubator (Thermo Fisher, USA) as described (Keyaerts et al., 2005). Screening against the Mu variant was not pursued further due to its poor adaptation to *in vitro* conditions.

### 2.2 Genomic sequencing

The complete genomes of the three variants of the SARS-CoV-2 virus circulating in Panama, A2.5 (M2169), Mu (SEQ83), and Delta (SEQ203), were obtained by next-generation sequencing (NGS) using the MySeq equipment (Illumina, San Diego, CA, USA). The amino acid (aa) sequences of the S protein (surface glycoprotein, 1,273 aa) and N protein (nucleocapsid, 420 aa) of the human SARS CoV-2 were then compared between the A2.5, Mu and Delta variants relative to the reference variant Wuhan-Hu-1 (NC04551.2). The alignments were made using the Ugene program version 44.0 under the default parameters of the MUSCLE application.

### 2.3 Virus titration

Using the plaque assay as described (Mendoza et al., 2020), we calculated the viral titers and expressed them as the number of plaque-forming units x mL (PFU x ml^-1^) (Baer and Kehn-Hall, 2014). Briefly, 2.0 × 10^5^ cells/well were seeded in 6-well dishes and incubated at 37°C, 5% CO_2_ overnight. When the plates were sub-confluent, tenfold serial dilutions of the stock of each SARS-CoV-2 strain were added in 150 µL of maintenance media. After incubation, 2 mL of overlay (MEM 2x + gentamicin 50 μg, FBS 1%, Sea plaque 2%) was added directly to each well and incubated further at 37°C, 5% CO_2_ for 72 h. At the end of incubation, 1 mL of 10% formaldehyde was added to the plate and incubated at room temperature for 1–2 h, followed by two washes with water before staining with 200 µL of 0.2% crystal violet. Finally, the plaque count was carried out.

### 2.4 Compounds

Twenty-six antimalarial, antiparasitic, and antiviral compounds (Supplementary Table S1) were screened for antiviral activity against the Delta, Mu, and A2.5 variants of the SARS-CoV-2 virus isolated in Panama during 2020–2022. To avoid selection bias, the compounds were coded and anonymized. Briefly, each compound was re-suspended in dimethyl sulfoxide (DMSO) (Sigma Aldrich, St. Louis, USA) or ddH20 and diluted in MEM or Eagles Minimum Essential Medium (EMEM) (BioWhittaker^®^) (Lonza, Walkersville, MD, USA), to a 500 µM concentration.

**TABLE 1:** Summary of mutations detected in the S protein of variants of the SARS-Cov-2 virus circulating in Panama during 2020–2022.

SARS-Cov-2 Mutations											
AA Position	19	95	137-143	144	156-158	216-219	456	482	505	618	685
Wuhan (Ref)`	T	T	NDPFLGV	—	EFR	—	L	T	N	D	P
A2.5	T	T	NDPFLGV	—	EFR	AAGYins	L456R	T	N	D618G	P
Mu	T	T95I	NDPFLGV	144Tins	EFR	—	L	T	N505Y	D618G	P685H
Delta	T19R	T	137-143del	—	156-158del	—	L456R	T482K	N	D618G	P685R

AA, Aminoacid; del, deletion; ins, insertion.

### 2.5 Cytotoxicity

To determine the maximum non-cytotoxic concentration (MNCC) of the compounds, seven 2-fold dilutions, starting at 250 μM, were dispensed in triplicate into a 96-well microplate just before the seeding of 1.5 × 10^4^ cells for Vero-E6 and 8.0 × 10^4^ cells for Calu-3 cells. The plates were then incubated for 48 h, followed by cytotoxicity testing using the methyl thiazolyl tetrazolium (MTT) assay or the CellTiter-Glo Luminescent Cell Viability Assay (Promega, Madison, WI, USA). The MNCC was defined as the maximum concentration of the compound that did not exert a significant cytotoxic effect compared to the control cells (Li et al., 2005), with the percent viability normalized to the untreated control cells. An 80% viability threshold was established for selecting the compounds for further analysis.

### 2.6 Viral load

To determine the viral copy numbers for each test well at 48 h post-infection (PI), we first extracted and purified the viral RNA from the supernatant using the MagMAX™ Viral/Pathogen Nucleic Acid Isolation Kit (Applied Biosystems, by Thermo Fisher Scientific, USA) with a KingFisher Flex Purification Automate machine (Life Technologies, Thermoscientific, Singapore). Then, we used a qRT-PCR AgPath-ID™ One-Step RT-PCR (Applied Biosystems, USA) with primers and probes for the specific amplification and detection of the viral nucleoprotein (N) of the SARS-CoV-2 virus using a QuantStudio 5 Real-Time PCR system (Applied Biosystems, USA). All assays were carried out in triplicate using the standard curve method with SARS-CoV-2 cDNA (Emery et al., 2004). The PCR program consisted of 50 cycles of 10 s at 95 C, annealing of 10 s at 58 C, and elongation of 10 s at 72 C to detect the N gene of SARS-CoV2. The following primers and probes were used: Primer Reverse. 5′-ATA​TTG​CAG​CAG​TAC​GCA​CAC​A- 3'(22 mer); Primer Forward. 5′-ACA​GGT​ACG​TTA​ATA​GTT​AAT​AGC​GT- 3'(26 mer); Primer Probe. 5′-ACA​CTA​GCC​ATC​CTT​ACT​GCG​CTT​CG-3' (26 mer).

### 2.7 Virus inhibition assay

The antiviral activity of the compounds was assessed by quantifying the viral load using an RT-qPCR assay. Briefly, the assay was performed in 48-well plates, where 80,000 cells per well were seeded using 500 µL of growth medium, quantifying the viral replication in the presence of various concentrations of the test compounds. Upon reaching 80% confluence, cells were washed with 500 µL of PBS (1x), and 100 µL of maintenance medium was added. The cells were then incubated with 100 µL of the culture medium solution containing the virus at a multiplicity of infection (MOI) of 0.00001 or 0.01, and the dish was incubated at 37°C with 5% CO2 for 1 hour. After 1 hour of incubation, the virus was removed, and the compound was diluted to its MNCC (Supplementary Table S1) before being added to 500 µL of culture medium. The appropriate controls (cells without virus and compounds, cells with virus alone, and cells with compounds) were included, and the microplate was incubated for 48 h at 37°C, 5% CO_2_. Assays were performed in triplicate. After 48 h, the supernatants from each well containing the viral load were collected for extraction and amplification.

### 2.8 IC_50_, CC_50,_ Cmax/EC_50_ and Cmax/EC_90_

To determine the half-maximal inhibitory concentration 50% (IC_50_) and half-maximal cytotoxic concentration 50% (CC_50_), sub-confluent monolayers of Vero E6 cells in 96-well plates were infected with the A2.5 variant at a multiplicity of infection (MOI) of 0.01. They were treated with seven two-fold dilutions of selected compounds in triplicate, starting with a concentration of 500 μM, and their percentage inhibition was calculated relative to the Dimethylsulfoxide (DMSO) control 72 h post-infection (PI) (Keyaerts et al., 2004).

The selectivity index (SI) was calculated as the ratio of the concentration of the compound that reduced cell viability by 50% (CC_50_ or 50% cytotoxic concentration) to the concentration of the compound needed to inhibit the viral cytopathic effect by 50% of the control value (IC_50_ or 50% inhibitory concentration). An SI > 10% was considered the threshold for further drug evaluation *in vivo*. DMSO was used as a negative control. To calculate the IC_50_ and CC_50_, a 4-parameter nonlinear regression sigmoidal concentration-response function was undertaken using the Prism 10 software package (GraphPad, Boston, MA, USA).

To calculate the maximum blood concentration obtained from the literature for each drug at doses commonly administered in human treatments, we calculated the ratios Cmax/EC_50_ and Cmax/EC_90_, where Cmax represents the expected maximum blood concentration derived from the *in vitro* results (Arshad et al., 2020).

### 2.9 Pre- and post-exposure assay

To evaluate the pre- and post-exposure activity of selected antimalarial compounds against the A2.5 or Delta variants, sub-confluent monolayers of Vero E6 cells in 96-well plates were exposed 1-h pre or 1-h post-infection (PI) to CQ and PQ at their inhibitory concentrations 90% (IC_90_), 50% (IC_50_) and 10% (IC_10_) determined previously during co-exposure assays and infected with a MOI = 0.01 of SARS-CoV-2 variants as described (Keyaerts et al., 2004). 72 h PI cell supernatants were collected, viral RNA was extracted, and the antiviral activity was determined using the quantitative RT-qPCR as described above.

### 2.10 Time-of-addition assay

We carried out a Time-of-addition (TOA) experiment to identify the specific stage of the viral cycle at which CQ and PQ exert their inhibitory effect on the SARS-Cov2 A2.5 and Delta variants as described with modifications (Daelemans et al., 2011). For this purpose, sub-confluent monolayers of Vero E6 cells in 96-well plates were infected with SARS-CoV-2 at a MOI of 0.01, as described (Keyaerts et al., 2004). After 20 min of adsorption, the cell monolayers were washed five times with MEM, followed by the addition of CQ and PQ at a concentration 10-fold above their IC_50_ (CQ at 63 µM and PQ at 153 µM) in triplicate as described (Daelemans et al., 2011), at 1-h pre-infection (time −1), at the time of infection (time 0) and subsequently at 3, 5 and 8 h PI. Eight hours after the last drug addition (when the first viral cycle was completed), 100 µL of cell supernatants were collected, viral RNA was extracted, and the antiviral activity was determined using a quantitative RT-qPCR as described above.

### 2.11 Western blot

To confirm the specificity of anti-SARS-CoV-2 antibodies against the N protein of the variants of the virus circulating in Panama, we used the Western blot (WB) technique (Towbin et al., 1979) using a Mini-PROTEAN vertical tetra electrophoresis system (Biorad, Hercules, CA) and a mouse antibody against SARS-CoV-2 nucleocapsid N protein (Abcam 281300).

### 2.12 Immunofluorescence

To confirm the infection of Vero-E6 cells infected with the Delta variant of the SARS-Cov2 virus, an indirect immunofluorescence (IFA) assay was designed as follows: Briefly, Vero-E6 cell monolayers infected with Delta variant at a MOI = 0.01 (1.2 × 10^6^ PFU/mL) and the uninfected negative control in an 8-well chamber mounted on a glass slide with sterile cover (Nunc Lab Tek II Chamber Slide System 154,534, Rochester, NY, USA) were incubated for 24 h in a CO_2_ atmosphere before fixation with 2% paraformaldehyde in PBS for 30 min, washed twice with PBS, permeabilized with 0.25% Triton X100 in PBS for 30 min, wash twice with PBS and block with 2% Skim Milk for 10 min. Subsequently, the rabbit anti-SARS-CoV-2 envelope primary antibody (abcam 272503) was incubated for 2 h in dilutions of 1/500, 1/1,000 and 1/2000 in duplicate and the secondary antibody goat anti-rabbit IgG conjugated with Alexa Fluor 647 (abcam150083) for 2 h in one line of the chamber in dilution of 1/800 and in the other 1/1,600 in a humid chamber protected from light at room temperature. The positive control was incubated with the anti-Nuc mouse primary antibody (abcam281300) and the FITC-conjugated rabbit secondary antibody anti-mouse IgG. The glass slides were covered with a cover slip and mounted with the Fluorshield™ mounting medium with DAPI (F6182-20ML, Sigma, USA), and examined and documented with an Olympus IX73 inverted epifluorescence microscope (Olympus, Tokyo, Japan) and cellSens software (Olympus, Tokyo, Japan).

### 2.13 Statistical analysis

Parametric and non-parametric statistical analyses were performed using the Prism 10.0 software package (Graphpad, Boston, MA, USA).

## 3 Results

### 3.1 Mutations in the S and N proteins of the SARS-Cov-2 variants

**FIGURE 1:**
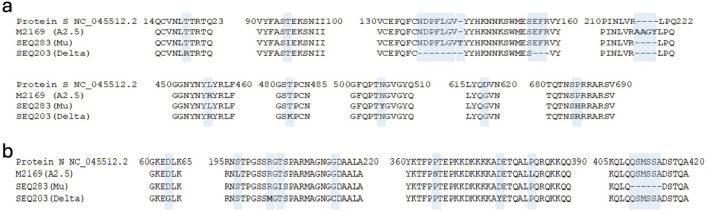
Amino acid mutations analysis of the S protein and N proteins of the SARS-CoV-2 variants. Alignment of amino acid sequences of the S **(a)** protein and the N **(b)** protein of SARS-Cov-2 of the A2.5, Mu, and Delta variants circulating in Panama with reference to the isolate Wuhan-Hu-1 (NC_045512.2).

Using the methodology described previously, we identified mutations (insertions, deletions, and substitutions) in the spike (S) and nucleocapsid (N) proteins of the A2.5, Mu, and Delta variants when compared against the reference isolate Wuhan-Hu-1 (NC_04551.2): The insertion 216AAGY219 and the substitution L456R in the A2.5 variant. The T95I substitution, the 144T insertion, and the N505Y and P685H replacement in the Mu variant. The T95I substitution, the 144T insert, T19R substitution, the N137-V143 and E156-R458 deletions, and substitutions L456R, T482K, and P685R in the Delta variant (Figure 1a; Table 1).

Regarding mutations in the N protein (nucleocapsid), which is composed of 420 aa with five domains that play a fundamental role in the viral cycle (V'Kovski et al., 2021) and is responsible for packaging the viral genome, we detected a series of mutations shown in Figure 1b and Table 1.

### 3.2 Titration of the SARS-Cov-2 variants by plaque assay in vero-E6 cells

**FIGURE 2:**
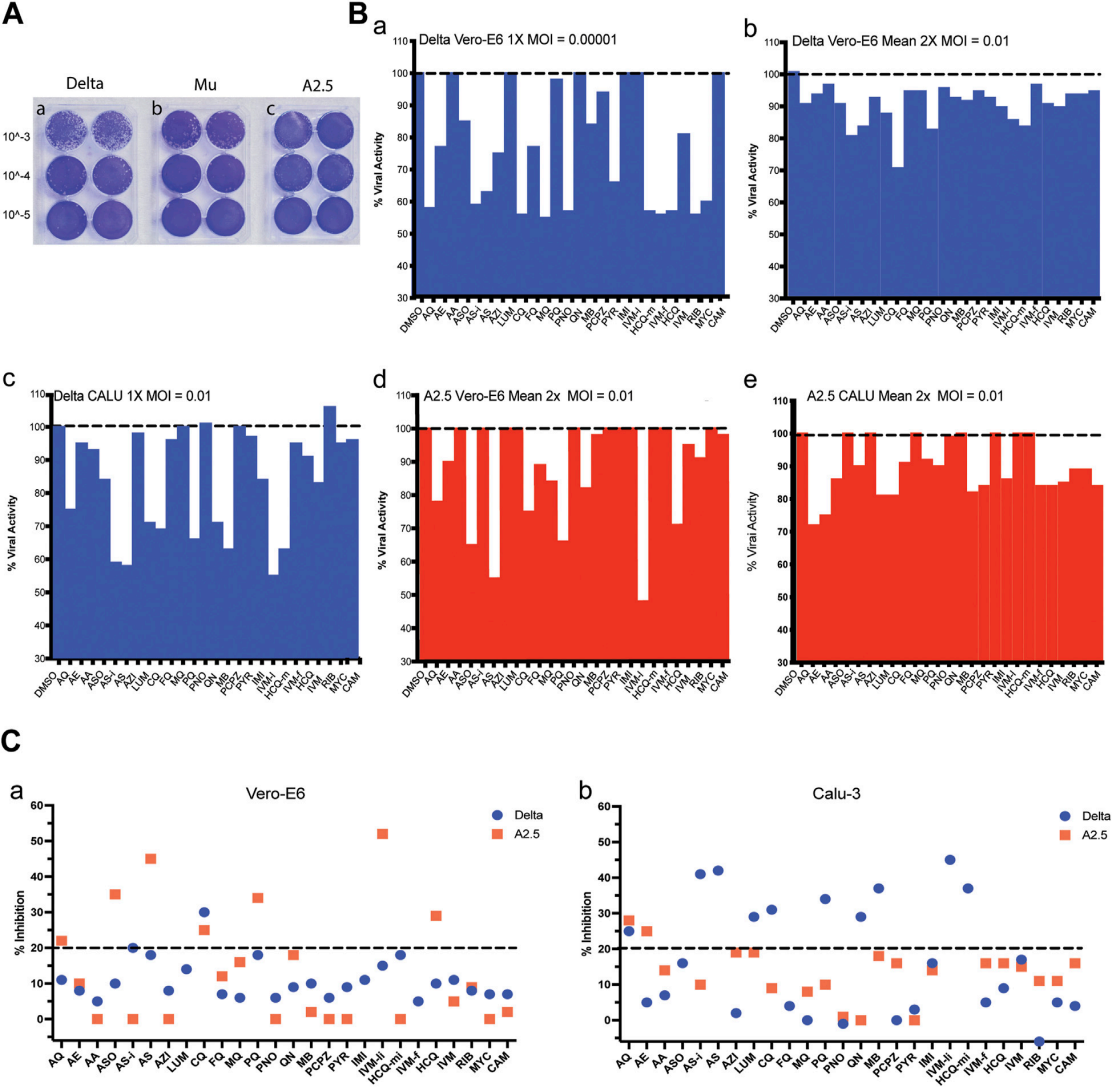
Viral titration and antiviral activity of antimalarial compounds against variants of the SARS-Cov-2 virus. **(A)** Virus titration by plaque forming units (PFU) was carried out in 6 well plates of Vero-E6 cells confluent monolayers infected with three dilutions in duplicate of viral stock (10^−3^ to 10^−5^) of the a) Delta, b) Mu, and c) A.2.5 variants of SARS-Cov2 isolated in Panama during 2020–2022. Titers were determined by counting the plaque number x reciprocal of dilution x reciprocal of the volume in mL expressed as PFU x ml^-1^. **(B)** a) Antiviral activity of 26 antimalarials and other compounds at their maximal non-cytotoxic concentration (MNCC) against the Delta and A2.5 variants in Vero-E6 and Calu-3 cells at a MOI = 0.00001. b-e) Antiviral activity at a MOI = 0.01. **(C)** Summary of the primary screening of antimalarial compounds against SARS-CoV-2 variants. a) Percentage of inhibition of active compounds against the Delta and A.2.5 variants in Vero-E6 cells. b) Percentage of inhibition of active compounds against the Delta and A.2.5 variants in Calu-3 cells. The black dashed line indicates the 20% viral inhibition threshold considered the selection limit equivalent to three standard deviations from the average inhibition of the DMSO control.

After 72 h of infecting the Vero-E6 cells monolayer, the Delta variant reached a viral titer of 3.5 × 10^6^ PFU/mL, the Mu variant 8. 5 × 10^5^ PFU/mL, and the A2.5 variant 4.0 × 10^6^ PFU/mL (Figure 2A).

### 3.3 Cytotoxicity and antiviral activity

The data summarized in Table 2 and Supplementary Table S1 show the cytotoxicity and antiviral activity results of the 26 antimalarials and other compounds evaluated against the SARS-CoV-2 virus variants at their MNCC. As shown, Vero-E6 cells did not reach the 80% viability threshold in 5 out of 26 (19%) of the compounds after 48 h of exposure, specifically artesunate (AS), ferroquine (FQ), quinine sulfate (QN), hydroxychloroquine-m (HCQ-m), and HCQ sulfate at their MNCC (Supplementary Figure S2a panel a). Similarly, Calu-3 cells did not achieve the 80% viability threshold in 5 out of 26 (19%) of the compounds after 48 h of exposure, specifically AS, pyronaridine (PNO), prochlorperazine (PCPZ), ivermectin-f (IVM-f), and HCQ sulfate (Supplementary Figure S2a panel b).

**TABLE 2:** Cytotoxicity and antiviral activity of selected antimalarials and other antiparasitic drugs in Vero-E6 and Calu-3 cells against the Delta and A2.5 variant of SARS-Cov2 isolated in Panama.

Cells	Class		% viability		% inhibition					
			Vero E6	Calu-3	Vero E6			Calu-3		
SARS-Cov2 variant					Delta	Delta	A2.5	Delta	A2.5	MNCC
MOI					0.0001	0.01	0.01	0.01	0.01	Concentration
Drug		Base MW	Mean 2x	Mean 2x	1x	Mean 2x	1x	1x	Mean 2x	uM
Amodiaquine (AQ)	4-aminoquinoline	395.4	84	82	42	11	22	25	28	20
Arteether (AE)	Sesquiterpene lactone	312.4	104	97	23	8	10	5	25	125
Artelinic Acid (AA)	Sesquiterpene lactone	418.5	100	92	0	5	0	7	14	30
Artemisone (ASO)	Sesquiterpene lactone	401.6	92	93	15	10	35	16	U	125
Artesunate injection (ASi)	Sesquiterpene lactone	384.5	81	82	41	20	0	41	10	50
Artesunic acid (AS)	Sesquiterpene lactone	384.4	74	74	37	18	45	42	U	125
Azithromycin dehydrate (AZH)	Macrolide antibiotic	749.0	92	87	25	8	0	2	19	30
Lumefantrine (LUM)	aryl amino alcohols	529.0	86	86	0	14	nd	29	19	250
Chloroquine diphosphate (CQ)	4-aminoquinoline	319.9	103	105	44	30	25	31	9	5
Ferroquine (FQ)	4-aminoquinoline	433.8	75	63	23	7	12	4	U	5
Mefloquine (MQ)	aryl amino alcohols	378.3	105	82	45	6	16	0	8	30
Primaquine Phosphate (PQ)	8-aminoquinoline	259.4	106	87	2	18	34	34	10	15
Pyranoridine (PNO)	Antifolate	518.1	91	78	43	6	0	-1	1	5
Quinine Sulfate (QN)	aryl amino alcohols	489.3	74	90	0	9	18	29	0	5
Methylene Blue Injection (MBi)	Oxidation-reduction agent	284.4	84	89	16	10	2	37	18	5
Prochlorperazine (PCPZ)	Phenothiazine derivative	373.9	80	77	6	6	0	0	16	30
Pyrimethamina (PYR)	Folic acid antagonist	248.7	94	92	34	9	0	3	0	125
Imidocarb (IMI)	Urea derivative	348.4	92	106	0	11	nd	16	14	53
Ivermectin Liquida (IVM-li)	Macrocycliclactone	875.1	85	87	0	15	52	45	U	30
Hydroxychloroquine Minsa (HCQ-m)	4-aminoquinoline	335.9	72	84	43	18	0	37	U	10
Ivermectin Farmacia (IVM-f)	Macrocycliclactone	875.1	101	67	44	5	nd	5	16	75
Hydroxychloroquine sulfate (HCQ)	4-aminoquinoline	335.9	47	64	43	10	29	9	16	62.5
Ivermectin (IVM)	Macrocycliclactone	875.1	93	87	19	11	5	17	15	8
Ribavirin (RIB)	Purine nucleoside analog	244.2	104	91	44	8	9	-6	11	250
Mycophenolic acid (MYC)	Immunosuppressant	320.3	107	109	40	7	0	5	11	5
Camostat mesylate (CAM)	Serine protease inhibitor	494.5	118	112	0	7	2	4	16	50

U, undetermined; nd, not determine.

When the antiviral activity (% inhibition) of the 26 compounds was screened at their MNCC in Vero-E6 cells at a MOI = 0.00001, eleven out of twenty-one (52%) of the compounds showed antiviral inhibition ≧ 20% with viability ≧ 80% for both variants, including amodiaquine (AQ), arteether (AE), artesunate injection (ASi), azithromycin (AZH), chloroquine (CQ), mefloquine (MQ), pyranoridine (PNO), pyrimethamine (PYR), ivermectin (IVM-f), ribavirin (RIB), mycophenolic acid (MYC) (Figure 2B panel a; Table 2). In contrast, at a MOI = 0.01 in Vero-E6 cells, only 2/21 (9%) of the compounds, including ASi and CQ showed viral inhibition ≧ 20% with viability ≧ 80% against the Delta variant (Figure 2B panel b; Figure 2C panel a), while 5/17 (29%) including amodiaquine AQ, ASO, CQ, PQ, and IVM-li showed antiviral inhibition ≧ 20% with viability ≧ 80% against the A2.5 variant (Figure 2B panel d; Figure 2C panel a); suggesting that their antiviral activity was SARS-CoV-2 variant dependent as well as viral dose dependent and there was a differential susceptibility to the antimalarials tested between the Delta and A2.5 variants at their MNCC.

Nevertheless, in Calu-3 cells infected with the Delta variant at a MOI = 0.01 nine out of twenty-one (42%) of the compounds examined, including AQ, AS-i, LUM, CQ, PQ, QN, MB, IVM-l, and HCQ-m, had viral inhibition ≧ 20% with viability ≧ 80% (Figure 2B panel c; Figure 2C panel b). Meanwhile, only 2 out of 10 (20%), including AQ and AE, showed viral inhibition ≧ 20% with viability ≧ 80% against the A2.5 variant in Calu-3 cells (Figure 2B panel e; Figure 2C panel b). These results suggest that besides the differential susceptibility between the SARS-CoV-2 variants tested, the antiviral activity of these compounds is also dependent on the host cell type.

**FIGURE 3:**
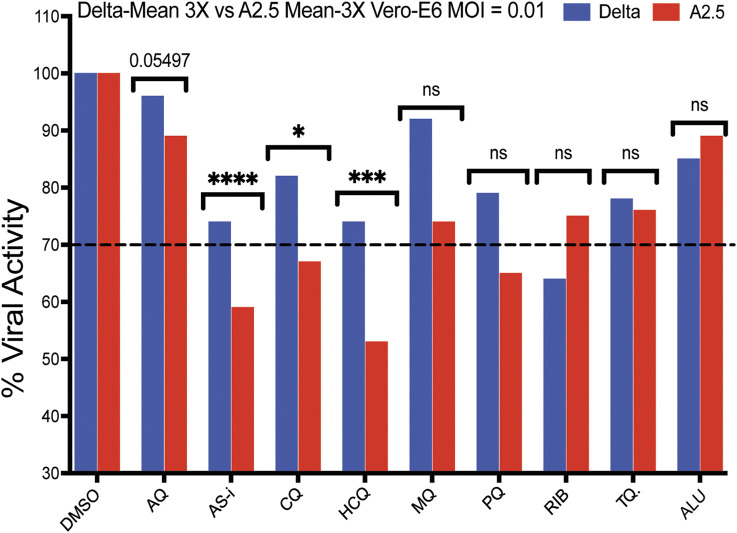
Antiviral activity of nine selected antimalarial compounds against variants of the SARS-CoV-2 virus. Antiviral activity against the Delta and A.2.5 variants in Vero-E6 cells. Bars indicate the mean viral load of three biological replicates (3X). Those compounds with a mean viral inhibition of ≥30% were selected for further analysis. Blue bars = Delta variant; Red bars = A2.5 variant. Multiple unpaired t-test statistical significance. * = P < 0.05; ** = P < 0.01; *** = P < 0.001; **** = P < 0.0001. ns = not significant. The antiviral activity of the compounds was determined using a qRT-PCR assay to quantify the viral load in infected Vero-E6 cells at a MOI = 0.01 relative to the non-treated controls. Tafenoquine (TQ), Artemisinin-Lumefantrine (ALU).

A secondary screening using Vero-E6 cells to confirm the antiviral activity and cytotoxicity of nine down-selected compounds against the Delta and A2.5 variants confirmed the differential antiviral activity of ASi, CQ, HCQ, PQ, and tafenoquine (TQ) (Figure 3), this time, using a threshold for viral inhibition ≧ 30%. A statistically significant difference in the mean viral inhibition between the Delta compared to the A2.5 variant was detected for ASi (t-test; p < 0.0001), CQ (t-test; p < 0.05), and HCQ (t-test; p < 0.001) (Figure 3). All three drugs being 20%, 20%, and 40% less potent against the Delta compared to the A2.5 variant, confirming the decreased susceptibility of the Delta variant to these compounds. In this experiment, a generic QHS/LUM combination tablet (OXALIS lab, Himachal Pradesh, India) containing 20 mg of QHS and 120 mg of LUM (ALU) did not show viral inhibition ≧ of 20% for neither the Delta nor the A2.5 variants (Figure 3). This time, the cytotoxicity of the compounds, except for HCQ, passed the 80% threshold, as shown in Supplementary Figure S3.

A heat map shown in Supplementary Figure S2b summarizes viral inhibition % in each of the four-virus variant host cell combinations, with the Delta-Calu-3 combination being the most sensitive with nine of twelve (75%) of the compounds exhibiting viral inhibition ≧ 20%; and IVM-li displaying the highest viral inhibition, 45% in the Delta-Calu-3, and 52% in the A2.5-Vero-E6 combinations, the highest detected in the study.

### 3.4 Selectivity index, inhibitory concentration 50% (IC_50_), and cytotoxic concentration 50% (CC_50_), Cmax/IC_50_ and Cmax/IC_90_

**FIGURE 4:**
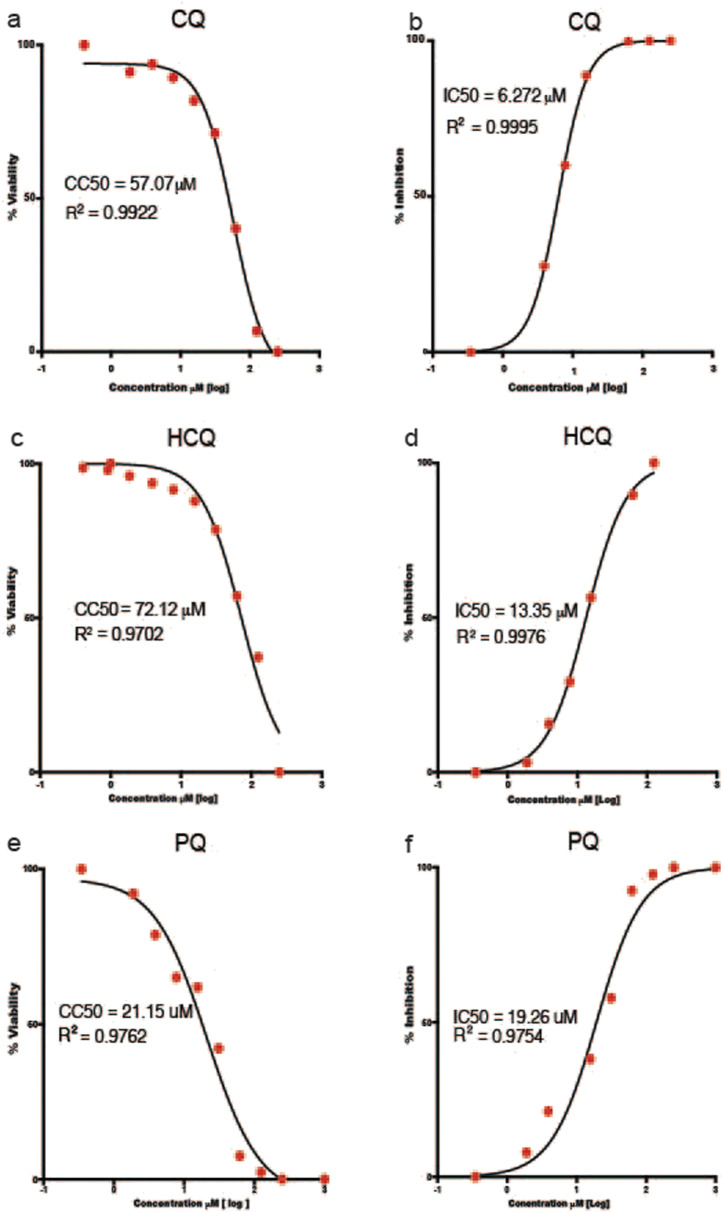
Half Maximal Cytotoxic Concentration 50% (CC_50_) and Half Maximal Inhibitory Concentration 50% (IC_50_) of selected antimalarial compounds. **(a,b)**)chloroquine (CQ); **(c,d)** hydroxychloroquine (HCQ); and **(e,f)** primaquine (PQ) in Vero-E6 cells against the A.2.5 variant of SARS-CoV-2 at a MOI = 0.01. Sigmoidal 4 PL, X is log(Concentration), least square fit.

The results of the IC_50_, CC_50_, Cmax/**IC**_**50**_ and Cmax/**IC**_**90**_ and selectivity index in Vero-E6 cells infected with the A.2.5 variant of SARS-CoV-2 at a MOI = 0.01 are presented in Table 3 and Figure 4. The cells were treated with a series of double dilutions of compounds, including ASi, CQ, HCQ, IVM, and PQ, starting from concentrations of 250–1,000 µM. All the compounds showed selectivity indices below 10, with CQ having the highest value, indicated by a selectivity index of 8, a Cmax/IC_50_ of 1.35, and a Cmax/IC_90_ ratio of 0.14. For calculations, Cmax information was extracted from the literature on ASi (Byakika-Kibwika et al., 2012), CQ, HCQ (Nicol et al., 2020), IVM (Gonzalez Canga et al., 2008), and PQ (Bhatia et al., 1986).

**TABLE 3:** Selectivity index of antimalarial drugs with viral inhibition activity ≥30% against the A2.5 variant of SARS-CoV-2 in Vero E6 cells.

Compound	IC50% μM	CI95%	µg/mL	CC50% µM	CI95%	µg/mL	IC90% μM	µg/mL	Cmax µg/mL	C_max_/IC50	C_max_/IC90	SI
Artesunate injection (Asi)	28.7	(20.9–39.6)	11.0	70.3	(68.3–72.5)	27.0	258.3	99.3	3.30	0.30	0.03	2
*Chloroquine sulfate (CQ)	6.3	(6.0–6.5)	2.0	53.2	(42.2–73.7)	17.0	56.7	18.1	2.70	1.35	0.14	8
*Hydroxychloroquine sulfate (HCQ)	12.6	(11.0–14.5)	5.8	72.1	(57.6–89.7)	23.2	113.4	38.1	0.13	0.02	3.00E-03	6
Ivermectin (IVM)	72.7	(58.8–103.8)	63.6	74.0	(39.2–137.7)	64.8	654.3	572.6	0.05	7.00E-04	8.70E-05	1
*Primaquine (PQ)	15.3	(9.0–15.0)	5.0	21.2	(10.1–38.0)	5.5	137.7	35.7	0.05	0.01	1.00E-03	1

IC50%, half-maximal inhibitory concentration 50%; CC50%, half-maximal cytotoxic concentration 50%; IC90%, inhibitory concentration 90%; CI95%, confidence inteval 95%; Cmax, maximum plasma concentration; SI, selectivity index = CC50%/IC50%.

*Mean of two biological replicates.

### 3.5 Pre- and post-infection assay

**FIGURE 5:**
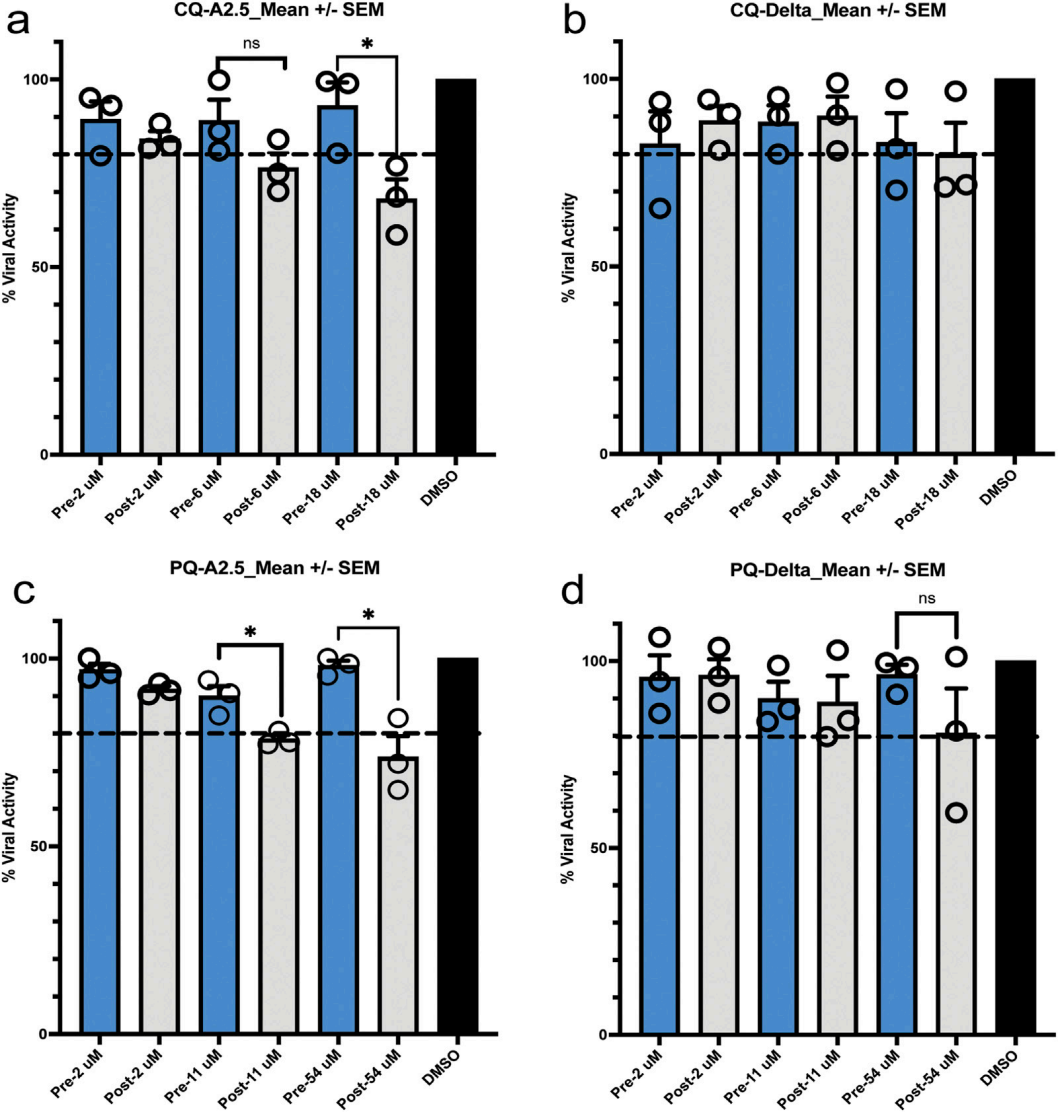
Pre- and post-exposure antiviral activity in Vero-E6 cells of chloroquine (CQ) and primaquine (PQ) cells to their IC_90_, CI _50_, and IC_10_ against SARS-CoV-2 variants isolated in Panama. **(a,b)** Pre- and post-infection antiviral activity of CQ against the A2.5 and Delta variants; **(c,d)** Pre- and post-infection antiviral activity of PQ against the A2.5 and Delta variants. Vero-E6 cells were exposed to indicated μM concentrations of CQ and PQ 1 hour before (pre-infection) (blue column) or 1 hour after infection (post-infection) (gray column) with the A2.5 and Delta variants at MOI = 0.01. The dashed line indicates the threshold of 20%, indicative of the selection limit. The black circles indicate the percentage of antiviral activity in each biological replicate. *=p < 0.05. ns = not significant.

Pre-treatment of Vero E6 cells with CQ or PQ 1 hour pre-infection at their inhibitory concentration 10% (IC_10_), 50% (IC_50_), and 90% (IC_90_) did not influence the viral replication of the A.2.5 or the Delta variants at a MOI = 0.01. In contrast, if treated 1 hour PI with CQ or PQ, a dose-dependent antiviral effect was observed against the A2.5 and Delta variants (Figure 5).

### 3.6 Time of drug addition assay

**FIGURE 6:**
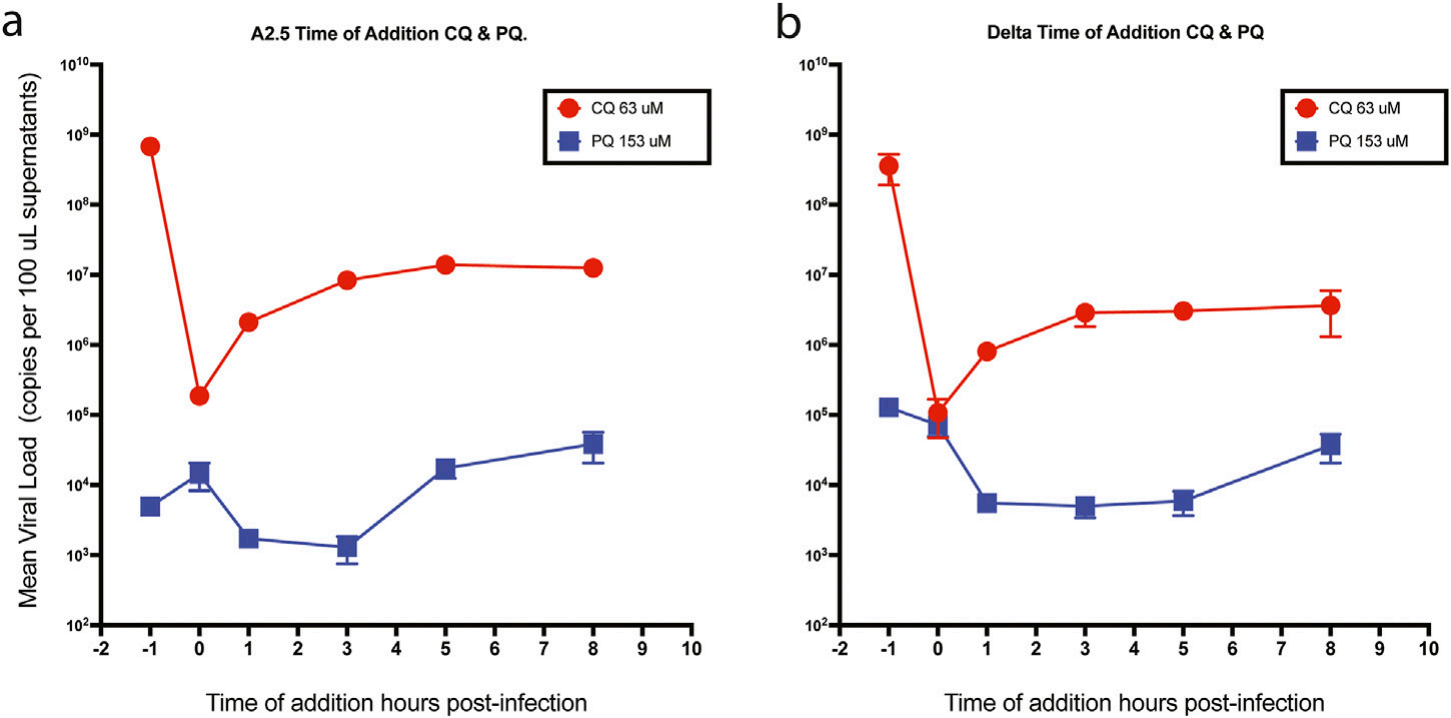
Time of drug addition. Mean viral loads of the A2.5 and Delta variants of SARS-CoV-2 in Vero-E6 cells treated with chloroquine (CQ) (red dots) and primaquine (PQ) (blue squares). **(a)** A2.5 **(b)** Delta. CQ (red dots) and PQ (blue squares) were added at 10-fold their IC50 at −1, 0, 3, 5, and 8 h of infection. Viral load = Mean ± SEM copies x 100 µL of supernatants. MOI = 0.01.

When CQ and PQ were added to Vero-E6 cells at a concentration 10-fold above their IC_50_ 1-h pre-infection (time −1 h), or CQ at a concentration of 63 μM, they did not show an effect on the viral load of neither the A.2.5 nor the Delta variants (Figure 6 panels a and b), suggesting that the drug has no antiviral effect. In contrast, compared to CQ, a substantially lower viral load of the A.2.5 and Delta variants was detected when treated with PQ at 153 μM, tenfold its IC_50_, suggesting that PQ had an antiviral effect at this dose level. Most of the antiviral effect of CQ occurred during the adsorption phase, with a decreasing effect during the first 3 h of the replication cycle, corresponding to uncoating and fusion in both the A.2.5 and the Delta variants. Conversely, PQ exerted most of its inhibitory effect during the first 3 h in the A2.5 and the first 5 h in the Delta variant, suggesting that the drug interferes with the adsorption and replication phases of the virus.

### 3.7 Epifluorescence and Western blot

**FIGURE 7:**
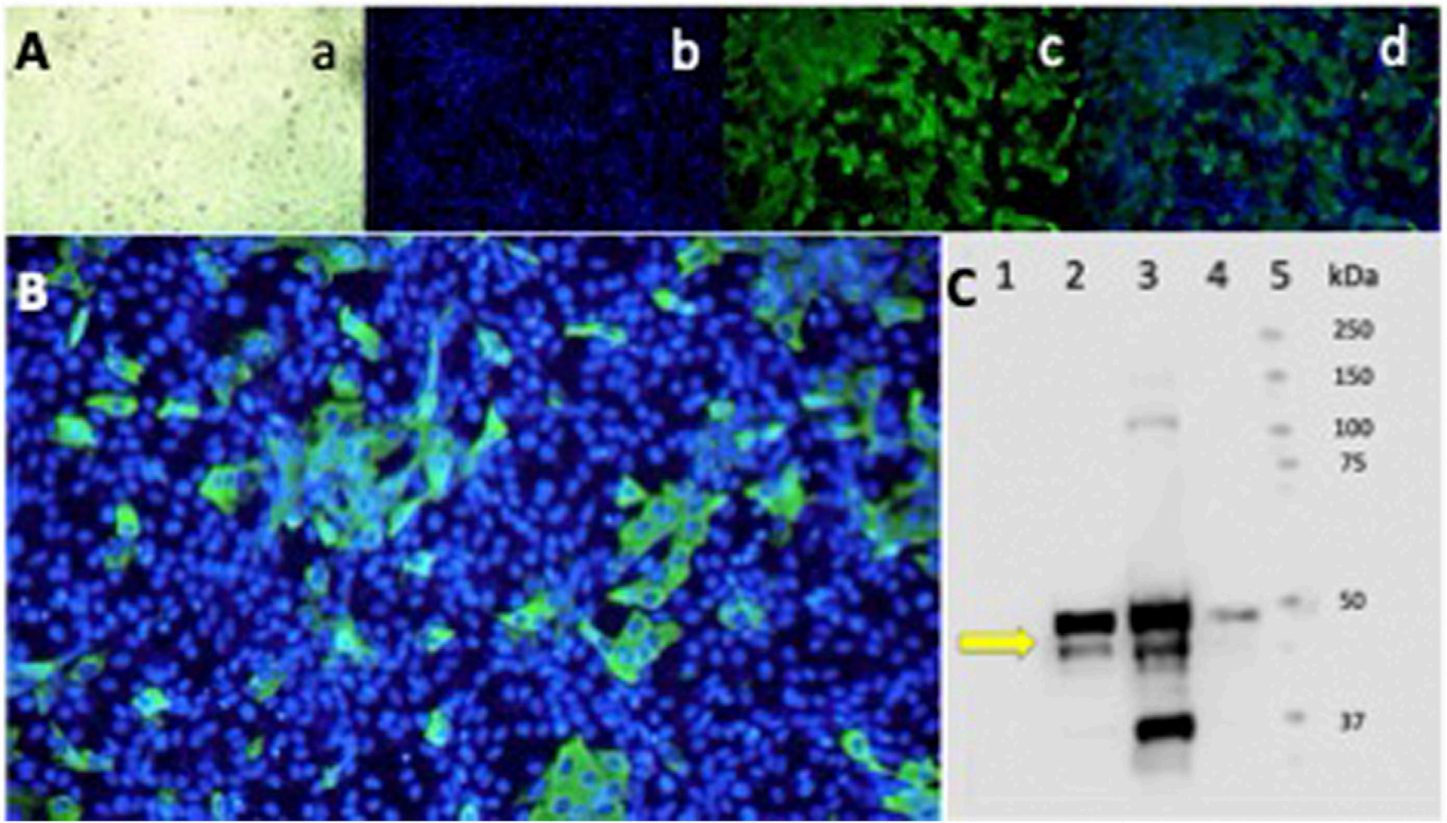
Epifluorescence and Western blot of Vero-E6 cells infected with the SARS-CoV-2 Delta variant. Indirect IFA in Vero-E6 cells infected with the Delta variant of SARS-CoV-2 at a MOI = 0.01 (1.2 × 10^−6^ PFU/mL) and incubated for 24 h in a CO2 atmosphere. **(A)** Epifluorescence images: a) light microscopy, b) DAPI, c) FITC, d) Merge 20X. **(B)** Merge 40X. Primary Ab against the N protein. Olympus IX73 inverted epifluorescence microscope (Tokyo, Japan). **(C)** Western blot analysis of Vero-E6 cell extracts infected with the SARS-CoV-2 Delta variant virus at different MOI and infection times. Lane: 1) Cell control. 2) Virus control + DMSO at 24 h of infection MOI 0.01. 3) Virus control + DMSO at 48 h of infection MOI 0.01. 4) Virus control at 48 h of infection MOI 0.00001. The antibody detects the N protein of SARS-CoV-2 at 55 kDa (yellow arrow). 5) Molecular weight markers.

To confirm the infection with the SARS-CoV-2 virus in infected Vero-E6 cells, the N protein was characterized as a diffuse cytoplasmic green fluorescence staining detected by epifluorescence (Figures 7A,B). A 55 kDa band, corresponding to the SARS-CoV-2 N protein, was detected by Western blot, confirming the infection of the Vero-E6 cells and the active production of new viral proteins (Figure 7C).

## 4 Discussion

Antimalarials of various classes, such as CQ and HCQ, have shown antiviral activity *in vitro* against the SARS-CoV-2 virus (Gendrot et al., 2020a; Ghosh et al., 2021; Gendrot et al., 2020b; Gendrot et al., 2020c). However, current evidence regarding their efficacy in clinical trials for treating or preventing COVID-19 is inconclusive (Gautret et al., 2020; Das et al., 2020). This uncertainty is primarily due to heterogeneous clinical study designs involving diverse study populations treated at different phases of the disease and infected with different SARS-CoV-2 variants (Zhou et al., 2023).

This *in vitro* study demonstrates the differential susceptibility to antimalarials of two SARS-CoV-2 variants isolated in Panama, A2.5, and Delta, when tested in Vero-E6 and Calu-3 cells. Our results suggest that the antiviral activity of the tested antimalarial compounds varies depending on the virus variant and the host cell used. Of the 26 antimalarials and other antiparasitic compounds tested initially, only the aminoquinolines, artemisinin derivatives, and IVM showed antiviral activity *in vitro*.

It is known that antimalarials belonging to the 4-aminoquinolines, such as CQ and HCQ, can interfere with viral entry and replication by altering endosomal pH and preventing viral fusion with the host cell. Additionally, they affect the glycosylation of the ACE2 receptor, resulting in the Spike protein’s inability to bind to ACE2 (Yao et al., 2020; Banerjee et al., 2023).

Of the four aminoquinolines tested, CQ at a concentration of 5 µM demonstrated the highest activity, achieving ≥20% viral inhibition against the A2.5 and Delta variants in Vero E−6 cells, as well as against the Delta variant in Calu-3 cells but not the A2.5 (data not shown). Other studies have reported that CQ inhibits SARS-CoV-2 with an EC50 of 2.1 μM and an EC90 of 3.8 μM (Gendrot et al., 2020c). Here, HCQ, the second most effective aminoquinoline, was 1.8 times more potent against the A2.5 variant than the Delta variant. It is known that the Delta variant harbors the N501Y mutation on the RBD of the Spike protein, which enhances its binding affinity to the ACE2 receptor (Chen et al., 2022), which could alter its susceptibility to 4-aminoquinolines or other compounds that affect the ACE2 receptor compared to other SARS-CoV-2 variants.

As expected, CQ and HCQ effectively reduced the infectivity of the Delta and A.2.5 variants of SARS-CoV-2 in Vero-E6 cells (Wang et al., 2020; Liu et al., 2020). However, in Calu-3 cells, the inhibitory effects on the A.2.5 variant were less pronounced (Persoons et al., 2021), suggesting that CQ and HCQ may not effectively prevent the viral entry and replication of the A2.5 variant in this cell line, which is the only one capable of supporting SARS-CoV-2 replication. However, its lower infectivity rates compared to Vero cells, along with factors such as timing of administration or dosage, may have also influenced the effectiveness of CQ and HCQ against the A2.5 variant.

In this study, the viability of the host cells was lowest when treated with hydroxychloroquine (HCQ) compared to the other compounds. Research has shown that HCQ can affect lipid metabolism and induce endoplasmic reticulum (ER) stress, reducing cell viability. Specifically, the modulation of ER stress pathways and lipid metabolism by HCQ can negatively impact cell viability (Zhou et al., 2022).

The known interaction between the SARS-CoV-2 viral spike protein (S) and human ACE2 through the RBD, along with the TMPRSS2 extracellular protease domain that cleaves the S protein, triggers cell membrane fusion and facilitates the uptake of the virus, influencing the susceptibility of host cells to the virus. Our results indicate that the difference in viral permissivity between cell lines, specifically Vero E6 and Calu-3 cells, influences their susceptibility to the antiviral effects of the various compounds tested.

Furthermore, it has been noted that CQ does not inhibit infection with SARS-CoV-2 in the TMPRSS2-expressing human lung cell line Calu-3 (Baer and Kehn-Hall, 2014; Hoffmann et al., 2020). In this study, CQ was able to inhibit the A.2.5 variant infection in Calu-3 cells, although at a lower level (two-fold less) compared to the TMPRSS2 inhibitor camostat (CAM) (Dittmar et al., 2021).

Time-of-addition experiments showed that both SARS-CoV-2 variants were 2–4 orders of magnitude less susceptible to CQ in treated Vero-E6 cells than to PQ at the same time interval. Furthermore, these experiments confirmed that the Delta variant was less susceptible to PQ than the A.2.5 variant during the adsorption and initial stages of the replication cycle (0–3 h PI), once again suggesting an advantage of Delta against the 4-aminoquinolines CQ and PQ. Interestingly, TQ, an 8-aminoquinoline derivative of PQ known to inhibit the SARS-CoV-2 main protease (Mpro) and TMPRSS2 proteases (Chen et al., 2022), in our experiments demonstrated at least 20% viral inhibition against the Delta variant of SARS-CoV-2 in Vero-E6 cells.

Other compounds tested in this study, such as artemisinin, derived from the sweet wormwood plant (Artemisia annua), have been used as antimalarial agents for decades. In addition to its longstanding application in traditional Chinese medicine for treating intermittent fever and chills, artemisinin combination therapy (ACTs) is currently the gold standard for managing uncomplicated malaria globally due to its endoperoxide properties. However, resistance is rapidly developing in Asia and Africa (Muller et al., 2019; van Loon et al., 2022; Grossman et al., 2023). More recently, artemisinin derivatives have also demonstrated the ability to inhibit various viruses, including SARS-CoV-2. In this study, we found that the synthetic artemisinin derivative ASi, at its minimum non-cytotoxic concentration (MNCC) of 50 μM, was two to four times more potent than other synthetic artemisinins such as AE, AA, and ASO at their respective MNCCs when tested in Vero E6 cells and Calu-3 cells infected with the A.2.5 variant. This finding aligns with results reported in other studies compared to artemether (Zhou et al., 2021).

Moreover, this study shows that the antiparasitic IVM at 30 µM was the most potent compound against the A.2.5 variant in Vero-E6 cells and the Delta variant in Calu-3 cells, achieving viral inhibitions of 45%–52%, respectively. *In vitro* studies have demonstrated that IVM reduces the replication of the SARS-CoV-2 virus (Caly et al., 2020). Three clinical trials and two observational studies have shown that using ivermectin significantly reduces the risk of SARS-CoV-2 infection; however, no significant association was observed between ivermectin prophylaxis and prognostic clinical outcomes (Zhou et al., 2023). Furthermore, ivermectin significantly reduces the time to viral clearance in mild to moderate COVID-19 patients (Rago et al., 2023). Despite this, other clinical trials using a single dose of ivermectin (200 μg/kg) administered to mild-to-moderate COVID-19 patients demonstrated its inefficacy in decreasing the time to a negative RT-PCR test (Wada et al., 2023).

In this study, both ivermectin and artesunate exhibited low selectivity index values, which contrasts with numerous clinical reports suggesting that ivermectin is effective in treating SARS-CoV-2, review in (Sansone et al., 2024). Several factors may account for the low selectivity index of ivermectin and artesunate observed in experimental studies. Ivermectin may inhibit SARS-CoV-2 entry by disrupting importin-dependent nuclear transport of viral proteins, while artesunate, primarily an antimalarial, has shown potential effects on viral replication and inflammation (Zhou et al., 2021). However, a low SI *in vitro* does not necessarily rule out clinical efficacy. It is crucial to consider additional mechanisms—such as immune modulation and pharmacokinetics—as they may play a significant role in the therapeutic benefits of these drugs (Bessis et al., 2022).

Despite the observed *in vitro* antiviral activity of the tested antimalarial compounds against the SARS-CoV-2 variants A2.5 and Delta in Vero E6 and Calu-3 cells, CQ exhibited a selectivity index of 8 in Vero E6 cells. This index falls short of the minimum requirement of 10 for advancing to animal studies. Thus, the compounds tested under our conditions exhibited a low selectivity index, consistent with previous findings on the antiviral activity of compounds such avermectins and milbemycin in Vero cells, Calu-3 cells, and mice (Chable-Bessia et al., 2022). These results suggest that under the tested conditions, these compounds do not exhibit sufficient antiviral activity combined with high viability and low cytotoxicity to be prioritized as antiviral candidates. Therefore, they are unsuitable for further pre-clinical research in animal models or for selection in human clinical trials.

*In vitro* studies on antimalarial drugs against SARS-CoV-2 provide a pathway for antiviral research. However, we demonstrate that their antiviral activity depends on viral variants and host cells. Therefore, before selecting the best candidates for animal studies, we emphasize the necessity of conducting screenings and validations with at least two different viral variants and two distinct cell types in future antiviral *in vitro* studies. Furthermore, most evidence of antiviral activity from antimalarial drugs acquired from clinical trials thus far is of moderate quality in randomized trials and lower quality in observational studies (Zhou et al., 2023). Nonetheless, considering the virus continuous evolution, exploring antimalarials or other antiparasitics as antiviral agents deserves further evaluation.

### 4.1 Limitations of the study

There are several potential limitations to the study. First, we must emphasize that *in vitro* results often correlate poorly with *in vivo* efficacy and safety. Therefore, well-designed animal studies and clinical efficacy trials must corroborate the results obtained *in vitro*. Additionally, during the primary screening phase of the study, we observed significant technical variation, both within (intra-assay) and between (inter-assay) assays—a challenge that was difficult to control. To mitigate the technical variation noted in the secondary screen, we utilized at least three technical and three biological replicates to reconfirm the activity of each selected compound for further evaluation. Lastly, it is essential to note the challenges of working with Calu-3 cells, a delicate cell line that takes longer to reach confluency than Vero-E6 cells.

## 5 Conclusion

The study highlights the importance of selecting appropriate cell models for SARS-CoV-2 research, as different variants (Delta vs. A2.5) show varying drug susceptibilities depending on the host cell type. Vero-E6 cells, widely used for viral studies, lack TMPRSS2, leading to an over-reliance on endosomal entry pathways, while Calu-3 cells, a lung epithelial model, provide a more physiologically relevant system. Some compounds were effective in 1 cell type but not the other, emphasizing the need for multiple models to obtain a comprehensive antiviral profile. Researchers should consider viral entry mechanisms, drug mode of action, and physiological relevance when choosing a model. Additionally, organoids or primary human airway cells may offer more clinically relevant data, reducing discrepancies between *in vitro* and *in vivo* findings (Chiu et al., 2023).

## Data Availability

The original contributions presented in the study are included in the article/Supplementary Material, further inquiries can be directed to the corresponding author.

## Supplementary material

SUPPLEMENTARY FIGURE S1 Amino acid variations within the full length of the spike **(A)** and nucleocapsid **(B)** proteins of the A2.5, Mu, and Delta variants are shown in red squares, compared against the reference isolate Wuhan-Hu-1 (NC_04551.2).

SUPPLEMENTARY FIGURE S2a MTT cell viability assay. **(a)** Vero-E6 and **(b)** Calu-3 cells exposed to 26 antimalarials and other compounds at their maximal non-cytotoxic concentration (MNCC) for 48 h. The dashed line represents the 80% viability threshold for further analysis. Methyl thiazolyl tetrazolium (MTT).

SUPPLEMENTARY FIGURE S2b Heatmap of the % viral inhibition of antimalarials against variants of the SARS-CoV-2 virus in Vero-E6 y Calu-3 cells.

SUPPLEMENTARY FIGURE S3 Cytotoxicity of nine selected antimalarial compounds against variants of the SARS-CoV-2 virus. CellTiter-Glo luminescence viability assay of Vero-E6 cells exposed to nine selected antimalarial compounds at different concentrations.

## References

[B1] Arshad U. Pertinez H. Box H. Tatham L. Rajoli R. K. R. Curley P. (2020). Prioritization of anti-SARS-cov-2 drug repurposing opportunities based on plasma and target site concentrations derived from their established human pharmacokinetics. Clin. Pharmacol. Ther. 108, 775–790. 10.1002/cpt.1909 32438446 PMC7280633

[B2] Baer A. Kehn-Hall K. (2014). Viral concentration determination through plaque assays: using traditional and novel overlay systems. J. Vis. Exp., e52065. 10.3791/52065 25407402 PMC4255882

[B3] Banerjee S. Banerjee D. Singh A. Kumar S. Pooja D. Ram V. (2023). A clinical insight on new discovered molecules and repurposed drugs for the treatment of COVID-19. Vaccines (Basel) 11, 332. 10.3390/vaccines11020332 36851211 PMC9967525

[B4] Bauman J. L. Tisdale J. E. (2020). Chloroquine and hydroxychloroquine in the era of SARS - CoV2: caution on their cardiac toxicity. Pharmacotherapy 40, 387–388. 10.1002/phar.2387 32285489

[B5] Bessis D. Trouillet-Assant S. Secco L. P. Bardin N. Blanc B. Blatiere V. (2022). COVID-19 pandemic-associated chilblains: more links for SARS-CoV-2 and less evidence for high interferon type I systemic response. Br. J. Dermatol 187, 1032–1035. 10.1111/bjd.21820 35971922 PMC9538550

[B6] Bhatia S. C. Saraph Y. S. Revankar S. N. Doshi K. J. Bharucha E. D. Desai N. D. (1986). Pharmacokinetics of primaquine in patients with P. vivax malaria. Eur. J. Clin. Pharmacol. 31, 205–210. 10.1007/BF00606660 3542534

[B7] Boonyasuppayakorn S. Reichert E. D. Manzano M. Nagarajan K. Padmanabhan R. (2014). Amodiaquine, an antimalarial drug, inhibits dengue virus type 2 replication and infectivity. Antivir. Res. 106, 125–134. 10.1016/j.antiviral.2014.03.014 24680954 PMC4523242

[B8] Byakika-Kibwika P. Lamorde M. Mayito J. Nabukeera L. Mayanja-Kizza H. Katabira E. (2012). Pharmacokinetics and pharmacodynamics of intravenous artesunate during severe malaria treatment in Ugandan adults. Malar. J. 11, 132. 10.1186/1475-2875-11-132 22540954 PMC3489518

[B9] Caly L. Druce J. D. Catton M. G. Jans D. A. Wagstaff K. M. (2020). The FDA-approved drug ivermectin inhibits the replication of SARS-CoV-2 *in vitro* . Antivir. Res. 178, 104787. 10.1016/j.antiviral.2020.104787 32251768 PMC7129059

[B10] Carabelli A. M. Peacock T. P. Thorne L. G. Harvey W. T. Hughes J. Consortium C.-G. U. (2023). SARS-CoV-2 variant biology: immune escape, transmission and fitness. Nat. Rev. Microbiol. 21, 162–177. 10.1038/s41579-022-00841-7 36653446 PMC9847462

[B11] Chable-Bessia C. Boullé C. Neyret A. Swain J. Hénaut M. Merida P. (2022). Low selectivity indices of ivermectin and macrocyclic lactones on SARS-CoV-2 replication *in vitro* . COVID 2, 60–75. 10.3390/covid2010005

[B12] Chen Y. Yang W. H. Chen H. F. Huang L. M. Gao J. Y. Lin C. W. (2022). Tafenoquine and its derivatives as inhibitors for the severe acute respiratory syndrome coronavirus 2. J. Biol. Chem. 298, 101658. 10.1016/j.jbc.2022.101658 35101449 PMC8800562

[B13] Chiu M. C. Zhang S. Li C. Liu X. Yu Y. Huang J. (2023). Apical-out human airway organoids modeling SARS-CoV-2 infection. Viruses 15, 1166. 10.3390/v15051166 37243252 PMC10220522

[B14] Daelemans D. Pauwels R. De Clercq E. Pannecouque C. (2011). A time-of-drug addition approach to target identification of antiviral compounds. Nat. Protoc. 6, 925–933. 10.1038/nprot.2011.330 21637207 PMC7086561

[B15] Das R. R. Jaiswal N. Dev N. Jaiswal N. Naik S. S. Sankar J. (2020). Efficacy and safety of anti-malarial drugs (chloroquine and hydroxy-chloroquine) in treatment of COVID-19 infection: a systematic review and meta-analysis. Front. Med. (Lausanne) 7, 482. 10.3389/fmed.2020.00482 32850924 PMC7403461

[B16] Dittmar M. Lee J. S. Whig K. Segrist E. Li M. Kamalia B. (2021). Drug repurposing screens reveal cell-type-specific entry pathways and FDA-approved drugs active against SARS-Cov-2. Cell Rep. 35, 108959. 10.1016/j.celrep.2021.108959 33811811 PMC7985926

[B17] Emery S. L. Erdman D. D. Bowen M. D. Newton B. R. Winchell J. M. Meyer R. F. (2004). Real-time reverse transcription-polymerase chain reaction assay for SARS-associated coronavirus. Emerg. Infect. Dis. 10, 311–316. 10.3201/eid1002.030759 15030703 PMC3322901

[B18] Fan H. H. Wang L. Q. Liu W. L. An X. P. Liu Z. D. He X. Q. (2020). Repurposing of clinically approved drugs for treatment of coronavirus disease 2019 in a 2019-novel coronavirus-related coronavirus model. Chin. Med. J. Engl. 133, 1051–1056. 10.1097/CM9.0000000000000797 32149769 PMC7147283

[B19] Gao J. Tian Z. Yang X. (2020). Breakthrough: chloroquine phosphate has shown apparent efficacy in treatment of COVID-19 associated pneumonia in clinical studies. Biosci. Trends 14, 72–73. 10.5582/bst.2020.01047 32074550

[B20] Gautret P. Lagier J. C. Parola P. Hoang V. T. Meddeb L. Mailhe M. (2020). Hydroxychloroquine and azithromycin as a treatment of COVID-19: results of an open-label non-randomized clinical trial. Int. J. Antimicrob. Agents 56, 105949. 10.1016/j.ijantimicag.2020.105949 32205204 PMC7102549

[B21] Gendrot M. Andreani J. Boxberger M. Jardot P. Fonta I. Le Bideau M. (2020c). Antimalarial drugs inhibit the replication of SARS-CoV-2: an *in vitro* evaluation. Travel Med. Infect. Dis. 37, 101873. 10.1016/j.tmaid.2020.101873 32916297 PMC7477610

[B22] Gendrot M. Andreani J. Jardot P. Hutter S. Delandre O. Boxberger M. (2020b). *In vitro* antiviral activity of doxycycline against SARS-CoV-2. Molecules 25, 5064. 10.3390/molecules25215064 33142770 PMC7663271

[B23] Gendrot M. Duflot I. Boxberger M. Delandre O. Jardot P. Le Bideau M. (2020a). Antimalarial artemisinin-based combination therapies (ACT) and COVID-19 in Africa: *in vitro* inhibition of SARS-CoV-2 replication by mefloquine-artesunate. Int. J. Infect. Dis. 99, 437–440. 10.1016/j.ijid.2020.08.032 32805422 PMC7426697

[B24] Ghosh A. K. Miller H. Knox K. Kundu M. Henrickson K. J. Arav-Boger R. (2021). Inhibition of human coronaviruses by antimalarial peroxides. ACS Infect. Dis. 7, 1985–1995. 10.1021/acsinfecdis.1c00053 33783182

[B25] Gignoux E. Azman A. S. de Smet M. Azuma P. Massaquoi M. Job D. (2016). Effect of artesunate-amodiaquine on mortality related to Ebola virus disease. N. Engl. J. Med. 374, 23–32. 10.1056/NEJMoa1504605 26735991

[B26] Gonzalez Canga A. Sahagun Prieto A. M. Diez Liebana M. J. Fernandez Martinez N. Sierra Vega M. Garcia Vieitez J. J. (2008). The pharmacokinetics and interactions of ivermectin in humans--a mini-review. AAPS J. 10, 42–46. 10.1208/s12248-007-9000-9 18446504 PMC2751445

[B27] Grossman T. Vainer J. Paran Y. Studensky L. Manor U. Dzikowski R. (2023). Emergence of artemisinin-based combination treatment failure in patients returning from sub-Saharan Africa with P. falciparum malaria. J. Travel Med. 30, taad114. 10.1093/jtm/taad114 37606241

[B28] Ho T. C. Wang Y. H. Chen Y. L. Tsai W. C. Lee C. H. Chuang K. P. (2021). Chloroquine and hydroxychloroquine: efficacy in the treatment of the COVID-19. Pathogens 10, 217. 10.3390/pathogens10020217 33671315 PMC7922580

[B29] Hoffmann M. Mosbauer K. Hofmann-Winkler H. Kaul A. Kleine-Weber H. Kruger N. (2020). Chloroquine does not inhibit infection of human lung cells with SARS-CoV-2. Nature 585, 588–590. 10.1038/s41586-020-2575-3 32698190

[B30] Huang M. Tang T. (2020). Expert consensus on chloroquine phosphate for new coronavirus pneumonia. Chin. J. Tuberc. Respir. Dis. 43. 10.3760/cma.j.issn.1001-0939.2020.0019

[B31] Kapepula P. M. Kabengele J. K. Kingombe M. Van Bambeke F. Tulkens P. M. Sadiki Kishabongo A. (2020). Artemisia spp. Derivatives for COVID-19 treatment: anecdotal use, political hype, treatment potential, challenges, and road map to randomized clinical trials. Am. J. Trop. Med. Hyg. 103, 960–964. 10.4269/ajtmh.20-0820 32705976 PMC7470522

[B32] Keyaerts E. Li S. Vijgen L. Rysman E. Verbeeck J. Van Ranst M. (2009). Antiviral activity of chloroquine against human coronavirus OC43 infection in newborn mice. Antimicrob. Agents Chemother. 53, 3416–3421. 10.1128/AAC.01509-08 19506054 PMC2715625

[B33] Keyaerts E. Vijgen L. Maes P. Neyts J. Van Ranst M. (2004). *In vitro* inhibition of severe acute respiratory syndrome coronavirus by chloroquine. Biochem. Biophys. Res. Commun. 323, 264–268. 10.1016/j.bbrc.2004.08.085 15351731 PMC7092815

[B34] Keyaerts E. Vijgen L. Maes P. Neyts J. Van Ranst M. (2005). Growth kinetics of SARS-coronavirus in Vero E6 cells. Biochem. Biophys. Res. Commun. 329, 1147–1151. 10.1016/j.bbrc.2005.02.085 15752773 PMC7092881

[B35] Khoo S. H. FitzGerald R. Saunders G. Middleton C. Ahmad S. Edwards C. J. (2022). Molnupiravir versus placebo in unvaccinated and vaccinated patients with early SARS-CoV-2 infection in the UK (AGILE CST-2): a randomised, placebo-controlled, double-blind, phase 2 trial. Lancet Infect. Dis. 23, 183–195. 10.1016/S1473-3099(22)00644-2 36272432 PMC9662684

[B36] Li Y. But P. P. Ooi V. E. (2005). Antiviral activity and mode of action of caffeoylquinic acids from Schefflera heptaphylla (L.) Frodin. Antivir. Res. 68, 1–9. 10.1016/j.antiviral.2005.06.004 16140400

[B37] Liu J. Cao R. Xu M. Wang X. Zhang H. Hu H. (2020). Hydroxychloroquine, a less toxic derivative of chloroquine, is effective in inhibiting SARS-CoV-2 infection *in vitro* . Cell Discov. 6, 16. 10.1038/s41421-020-0156-0 32194981 PMC7078228

[B38] Ma K. C. Castro J. Lambrou A. S. Rose E. B. Cook P. W. Batra D. (2024). Genomic surveillance for SARS-CoV-2 variants: circulation of omicron XBB and JN.1 lineages - United States, may 2023-september 2024. MMWR Morb. Mortal. Wkly. Rep. 73, 938–945. 10.15585/mmwr.mm7342a1 39446667 PMC11500842

[B39] Mendoza E. J. Manguiat K. Wood H. Drebot M. (2020). Two detailed plaque assay protocols for the quantification of infectious SARS-CoV-2. Curr. Protoc. Microbiol. 57, ecpmc105. 10.1002/cpmc.105 32475066 PMC7300432

[B40] Mohanty S. S. Sahoo C. R. Padhy R. N. (2021). Targeting some enzymes with repurposing approved pharmaceutical drugs for expeditious antiviral approaches against newer strains of COVID-19. AAPS PharmSciTech 22, 214. 10.1208/s12249-021-02089-5 34378108 PMC8354522

[B41] Muller O. Lu G. Y. von Seidlein L. (2019). Geographic expansion of artemisinin resistance. J. Travel Med. 26, taz030. 10.1093/jtm/taz030 30995310

[B42] Najjar-Debbiny R. Gronich N. Weber G. Khoury J. Amar M. Stein N. (2022). Effectiveness of paxlovid in reducing severe coronavirus disease 2019 and mortality in high-risk patients. Clin. Infect. Dis. 76, e342–e349. 10.1093/cid/ciac443 PMC9214014 35653428

[B43] Nicol M. R. Joshi A. Rizk M. L. Sabato P. E. Savic R. M. Wesche D. (2020). Pharmacokinetics and pharmacological properties of chloroquine and hydroxychloroquine in the context of COVID-19 infection. Clin. Pharmacol. Ther. 108, 1135–1149. 10.1002/cpt.1993 32687630 PMC7404755

[B44] Obaldia N. 3rd Kotecka B. M. Edstein M. D. Haynes R. K. Fugmann B. Kyle D. E. (2009). Evaluation of artemisone combinations in Aotus monkeys infected with Plasmodium falciparum. Antimicrob. Agents Chemother. 53, 3592–3594. 10.1128/AAC.00471-09 19506062 PMC2715646

[B45] Obaldia N. 3rd Milhous W. K. Kyle D. E. (2018). Reversal of chloroquine resistance of Plasmodium vivax in Aotus monkeys. Antimicrob. Agents Chemother. 62(9), 005822-e618. 10.1128/AAC.00582-18 PMC6125511 29941642

[B46] Obaldia N. 3rd Rossan R. N. Cooper R. D. Kyle D. E. Nuzum E. O. Rieckmann K. H. (1997). WR 238605, chloroquine, and their combinations as blood schizonticides against a chloroquine-resistant strain of Plasmodium vivax in Aotus monkeys. Am. J. Trop. Med. Hyg. 56, 508–510. 10.4269/ajtmh.1997.56.508 9180599

[B47] PAHO (2023). COVID-19 no longer constitutes a public health emergency of international concern. on Pan American Health Organization. Available online at: https://www.paho.org/en/news/8-5-2023-covid-19-no-longer-constitutes-public-health-emergency-international-concern (Accessed December 1, 2025).

[B48] Persoons L. Vanderlinden E. Vangeel L. Wang X. Do N. D. T. Foo S. C. (2021). Broad spectrum anti-coronavirus activity of a series of anti-malaria quinoline analogues. Antivir. Res. 193, 105127. 10.1016/j.antiviral.2021.105127 34217752 PMC8247284

[B49] Pillat M. M. Kruger A. Guimaraes L. M. F. Lameu C. de Souza E. E. Wrenger C. (2020). Insights in chloroquine action: perspectives and implications in malaria and COVID-19. Cytom. A 97, 872–881. 10.1002/cyto.a.24190 PMC7404934 32686260

[B50] Rago Z. Toth B. Szalenko-Tokes A. Bella Z. Dembrovszky F. Farkas N. (2023). Results of a systematic review and meta-analysis of early studies on ivermectin in SARS-CoV-2 infection. Geroscience 45, 2179–2193. 10.1007/s11357-023-00756-y 36879183 PMC9988599

[B51] Sansone N. M. S. Boschiero M. N. Marson F. A. L. (2024). Efficacy of ivermectin, chloroquine/hydroxychloroquine, and azithromycin in managing COVID-19: a systematic review of phase III clinical trials. Biomedicines 12, 2206. 10.3390/biomedicines12102206 39457519 PMC11505156

[B52] Satarker S. Ahuja T. Banerjee M. E V. B. Dogra S. Agarwal T. (2020). Hydroxychloroquine in COVID-19: potential mechanism of action against SARS-CoV-2. Curr. Pharmacol. Rep. 6, 203–211. 10.1007/s40495-020-00231-8 32864299 PMC7443392

[B53] Shi Q. Xia F. Wang Q. Liao F. Guo Q. Xu C. (2022). Discovery and repurposing of artemisinin. Front. Med. 16, 1–9. 10.1007/s11684-021-0898-6 35290595 PMC8922983

[B54] Si L. Bai H. Rodas M. Cao W. Oh C. Y. Jiang A. (2021). A human-airway-on-a-chip for the rapid identification of candidate antiviral therapeutics and prophylactics. Nat. Biomed. Eng. 5, 815–829. 10.1038/s41551-021-00718-9 33941899 PMC8387338

[B55] Tang Y. Liu J. Zhang D. Xu Z. Ji J. Wen C. (2020). Cytokine storm in COVID-19: the current evidence and treatment strategies. Front. Immunol. 11, 1708. 10.3389/fimmu.2020.01708 32754163 PMC7365923

[B56] Towbin H. Staehelin T. Gordon J. (1979). Electrophoretic transfer of proteins from polyacrylamide gels to nitrocellulose sheets: procedure and some applications. Proc. Natl. Acad. Sci. U. S. A. 76, 4350–4354. 10.1073/pnas.76.9.4350 388439 PMC411572

[B57] van Loon W. Oliveira R. Bergmann C. Habarugira F. Ndoli J. Sendegeya A. (2022). *In vitro* confirmation of artemisinin resistance in Plasmodium falciparum from patient isolates, southern Rwanda, 2019. Emerg. Infect. Dis. 28, 852–855. 10.3201/eid2804.212269 35318931 PMC8962885

[B58] V'Kovski P. Kratzel A. Steiner S. Stalder H. Thiel V. (2021). Coronavirus biology and replication: implications for SARS-CoV-2. Nat. Rev. Microbiol. 19, 155–170. 10.1038/s41579-020-00468-6 33116300 PMC7592455

[B59] Wada T. Hibino M. Aono H. Kyoda S. Iwadate Y. Shishido E. (2023). Efficacy and safety of single-dose ivermectin in mild-to-moderate COVID-19: the double-blind, randomized, placebo-controlled CORVETTE-01 trial. Front. Med. (Lausanne) 10, 1139046. 10.3389/fmed.2023.1139046 37283627 PMC10240959

[B60] Wang M. Cao R. Zhang L. Yang X. Liu J. Xu M. (2020). Remdesivir and chloroquine effectively inhibit the recently emerged novel coronavirus (2019-nCoV) *in vitro* . Cell Res. 30, 269–271. 10.1038/s41422-020-0282-0 32020029 PMC7054408

[B61] WHO. (2023). Weekly epidemiological update on COVID-19 - 10 August 2023. Available online at: https://www.who.int/publications/m/item/weekly-epidemiological-update-on-covid-19---10-august-2023?adgroupsurvey={adgroupsurvey}&gclid=Cj0KCQiA35urBhDCARIsAOU7QwkFzL0D2f7n82-DdFEi_5zEkhogATTDtUmNlKJfc12yWDzIodKQTaYaAm1YEALw_wcB (Accessed November 29, 2025).

[B62] Yan V. C. Muller F. L. (2021). Why remdesivir failed: preclinical assumptions overestimate the clinical efficacy of remdesivir for COVID-19 and Ebola. Antimicrob. Agents Chemother. 65, e0111721. 10.1128/AAC.01117-21 34252308 PMC8448091

[B63] Yao X. Ye F. Zhang M. Cui C. Huang B. Niu P. (2020). *In vitro* antiviral activity and projection of optimized dosing design of hydroxychloroquine for the treatment of severe acute respiratory syndrome coronavirus 2 (SARS-CoV-2). Clin. Infect. Dis. 71, 732–739. 10.1093/cid/ciaa237 32150618 PMC7108130

[B64] Zhong L. Zhao Z. Peng X. Zou J. Yang S. (2022). Recent advances in small-molecular therapeutics for COVID-19. Precis. Clin. Med. 5, pbac024. 10.1093/pcmedi/pbac024 36268466 PMC9579963

[B65] Zhou G. Verweij S. Bijlsma M. J. de Vos S. Oude Rengerink K. Pasmooij A. M. G. (2023). Repurposed drug studies on the primary prevention of SARS-CoV-2 infection during the pandemic: systematic review and meta-analysis. BMJ Open Respir. Res. 10, e001674. 10.1136/bmjresp-2023-001674 PMC10462970 37640510

[B66] Zhou N. Liu Q. Wang X. He L. Zhang T. Zhou H. (2022). The combination of hydroxychloroquine and 2-deoxyglucose enhances apoptosis in breast cancer cells by blocking protective autophagy and sustaining endoplasmic reticulum stress. Cell Death Discov. 8, 286. 10.1038/s41420-022-01074-6 35690609 PMC9188615

[B67] Zhou Y. Gilmore K. Ramirez S. Settels E. Gammeltoft K. A. Pham L. V. (2021). *In vitro* efficacy of artemisinin-based treatments against SARS-CoV-2. Sci. Rep. 11, 14571. 10.1038/s41598-021-93361-y 34272426 PMC8285423

