# Supplementary figures and images for: Antimalarial compounds exhibit variant- and cell-type-specific activity against SARS-CoV-2 isolated in Panama
Source: Front Pharmacol. 2025 Jun 4;16:1537053. doi: 10.3389/fphar.2025.1537053 (PMC12175177; doi:10.3389/fphar.2025.1537053)

A

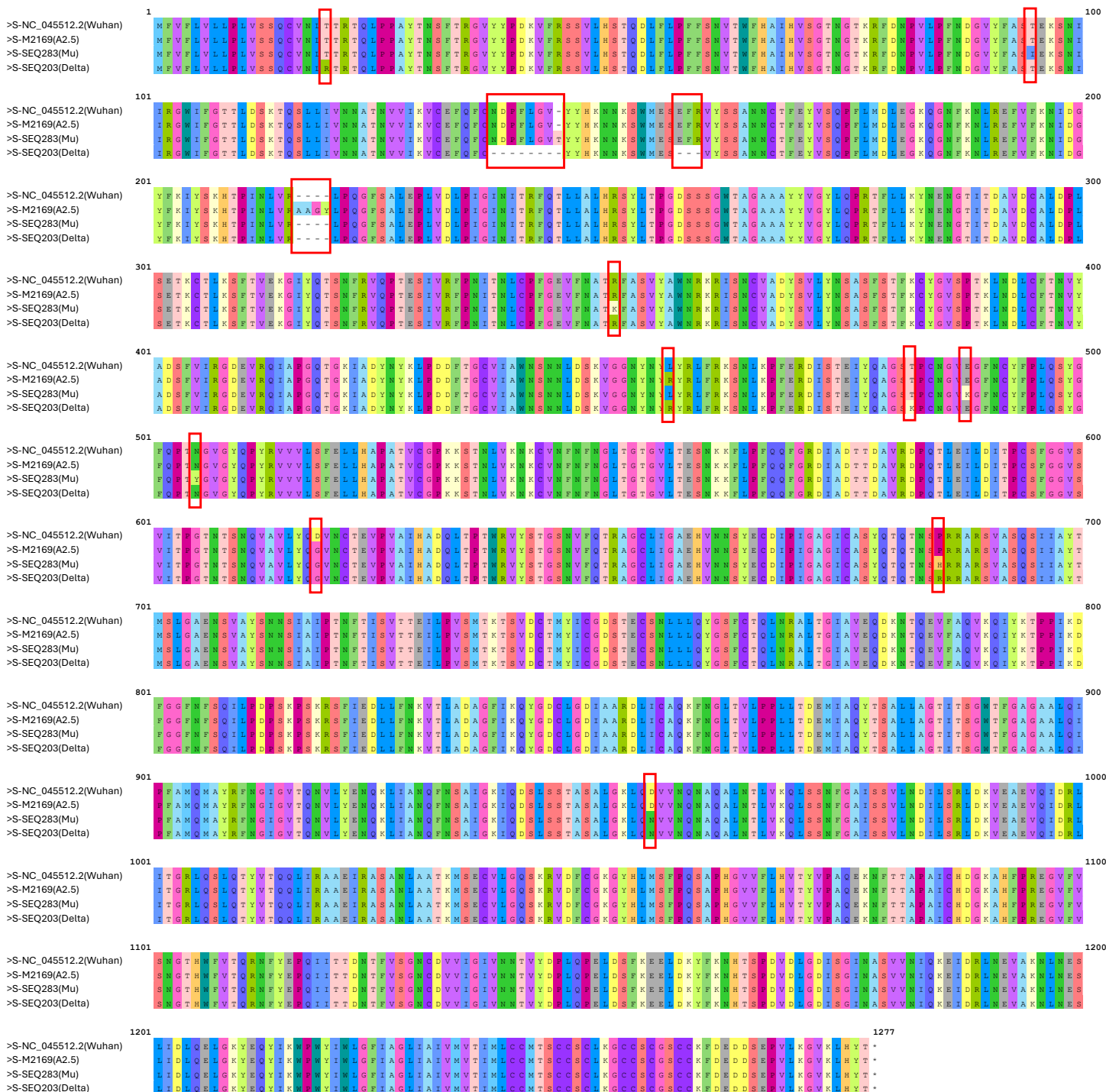

# B

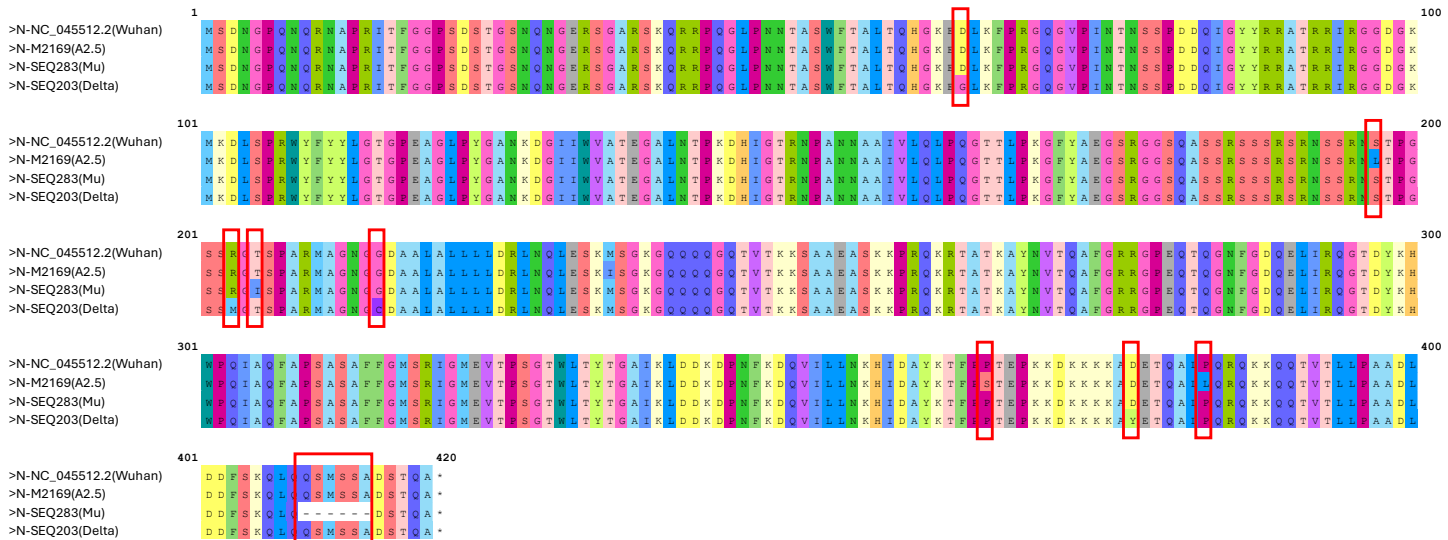

Supplement: Supplementary file 2 [file Supplementaryfile1.zip › Supplementary Figure S1.pdf]

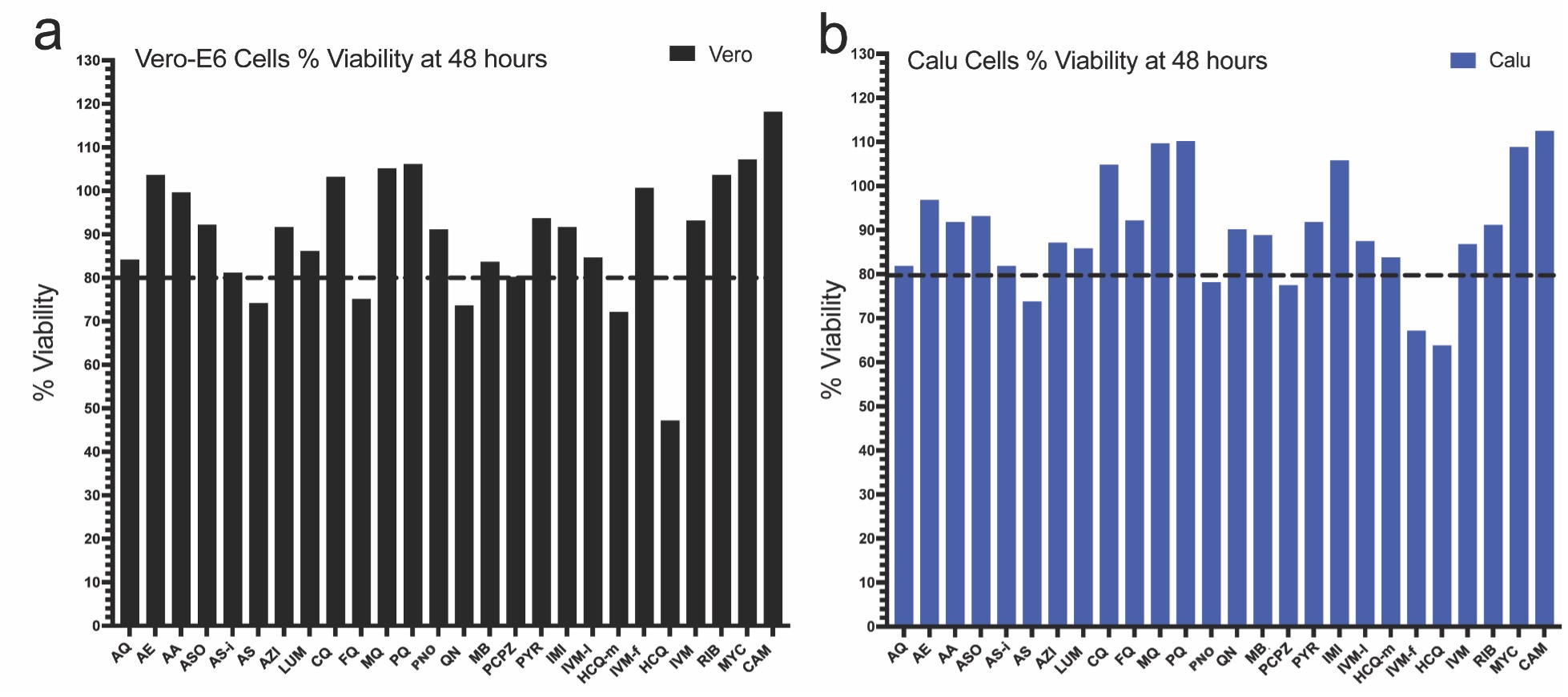

Supplement: Supplementary file 2 [file Supplementaryfile1.zip › Supplementary Figure S2a.jpg]

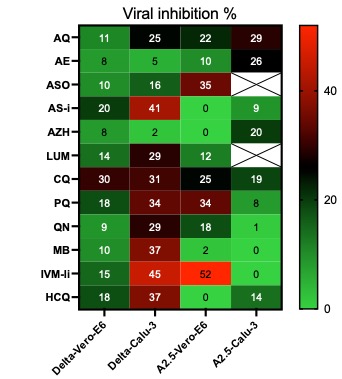

Supplement: Supplementary file 2 [file Supplementaryfile1.zip › Supplementary Figure S2b.jpg]

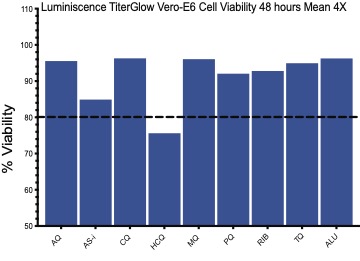

Supplement: Supplementary file 2 [file Supplementaryfile1.zip › Supplementary Figure S3.jpg]
